# Nasal carriage of *Staphylococcus pseudintermedius* in patients with granulomatosis with polyangiitis

**DOI:** 10.1093/rheumatology/key317

**Published:** 2018-11-08

**Authors:** Andreas Kronbichler, Beth Blane, Mark A Holmes, Josef Wagner, Julian Parkhill, Sharon J Peacock, David R W Jayne, Ewan M Harrison

**Affiliations:** 1 Vasculitis and Lupus Clinic, Addenbrooke’s Hospital, Cambridge, UK; 2 Department of Internal Medicine IV (Nephrology and Hypertension), Medical University Innsbruck, Innsbruck, Austria; 3 Department of Medicine, University of Cambridge, Addenbrooke’s Hospital, Cambridge, UK; 4 Department of Veterinary Medicine, University of Cambridge, Cambridge, UK; 5 Wellcome Trust Sanger Institute, Wellcome Genome Campus, Hinxton, UK; 6 Department of Pathogen Molecular Biology, London School of Hygiene and Tropical Medicine, London, UK

Rheumatology key message

- Patients with granulomatosis with polyangiitis can be colonized by *Staphylococcus pseudintermedius*, with unclear contribution to disease pathogenesis.

Sir, granulomatosis with polyangiitis (GPA, formerly Wegener’s granulomatosis) is characterized by necrotizing granulomatous inflammation usually involving the upper and lower respiratory tract, and necrotizing vasculitis affecting predominantly small to medium vessels, frequently leading to glomerulonephritis [1]. Disease etiopathogenesis is complex but includes a genetic background, epigenetic modifications and environmental factors. There are several lines of evidence indicating an association of nasal colonization with *Staphylococcus aureus* and GPA. *S. aureus* is an independent risk factor for relapse of GPA in carriers, and therapeutic administration of trimethoprim-sulfamethoxazole has been shown to reduce relapse rates during a treatment period of 2 years [2]. Little is known about other bacterial species that colonize the noses of GPA patients.

We undertook a study to investigate the bacterial species carried by 69 patients with a diagnosis of GPA and ENT involvement. This work was approved by the National Research Ethics Service (NRES) Committee East of England – Cambridge Central (REC reference: 08/H0308/176). Informed consent was obtained before sample collection. Nasal swabs (1–3 swabs per patient) were inoculated into high-salt (7.5%) nutrient broths and incubated statically at 37°C overnight; from this culture, 100 µl was inoculated onto Brilliance Staph 24 Agar (all media sourced from Oxoid, UK). Representative blue single colonies (putative *S. aureus*) were picked and streaked to purity on Columbia blood agar for further analysis. We noted that swabs from two patients (referred to as 0045 and 0093, neither with active disease in the ENT tract when sampled) grew blue colonies (see Supplementary Fig. S1A–D, available at *Rheumatology* online) identified as *Staphylococcus pseudintermedius* by matrix-assisted laser desorption ionization–time of flight mass spectrometry (MALDI-TOF MS, Bruker, Germany). Both were on immunosuppression (mycophenolate mofetil and rituximab alongside steroids, respectively) at the time of sampling (Supplementary Table S1, available at *Rheumatology* online). Patient 0045 was positive for *S. pseudintermedius* on three occasions spaced 6 weeks apart (confirming persistent carriage), but we only obtained a single swab from patient 0093.

Ten colonies from each of the primary culture plates positive for *S. pseudintermedius* (30 colonies total for 0045 and 10 for 0093) were submitted for whole-genome sequencing (Supplementary Table S2, available at *Rheumatology* online). Ten colonies were picked from each sample to determine whether the two participants carried single or multiple *S. pseudintermedius* clones, as previously reported for *S. aureus* [3]*.* Whole-genome sequence data confirmed the species identification. Multilocus sequence typing derived from the sequence data indicated that patient 0045 carried sequence type (ST) 155 strain in all three samples (see Fig. 1a), and generation of a core genome phylogeny revealed that the 30 isolates differed by a total of 138 single-nucleotide polymorphisms across the core genome. Patient 0093 carried a novel ST that was subsequently assigned as ST1025; the 10 isolates from this patient differed by a total of 13 single-nucleotide polymorphisms (see Fig. 1b). All isolates were genotypically methicillin susceptible on the basis of being *mecA* negative. Isolates from patient 0045 contained genes mediating resistance to penicillin (*blaZ*), tetracycline (*tetM*) and aminoglycosides (*aacA*-aphD), and 6 of 30 isolates tested from this case carried the trimethoprim-resistance gene *dfrG* (Fig. 1a). Isolates from patient 0093 were positive for *blaZ* (penicillin resistance) alone (Supplementary Table S2, available at *Rheumatology* online).

**Fig. 1:**
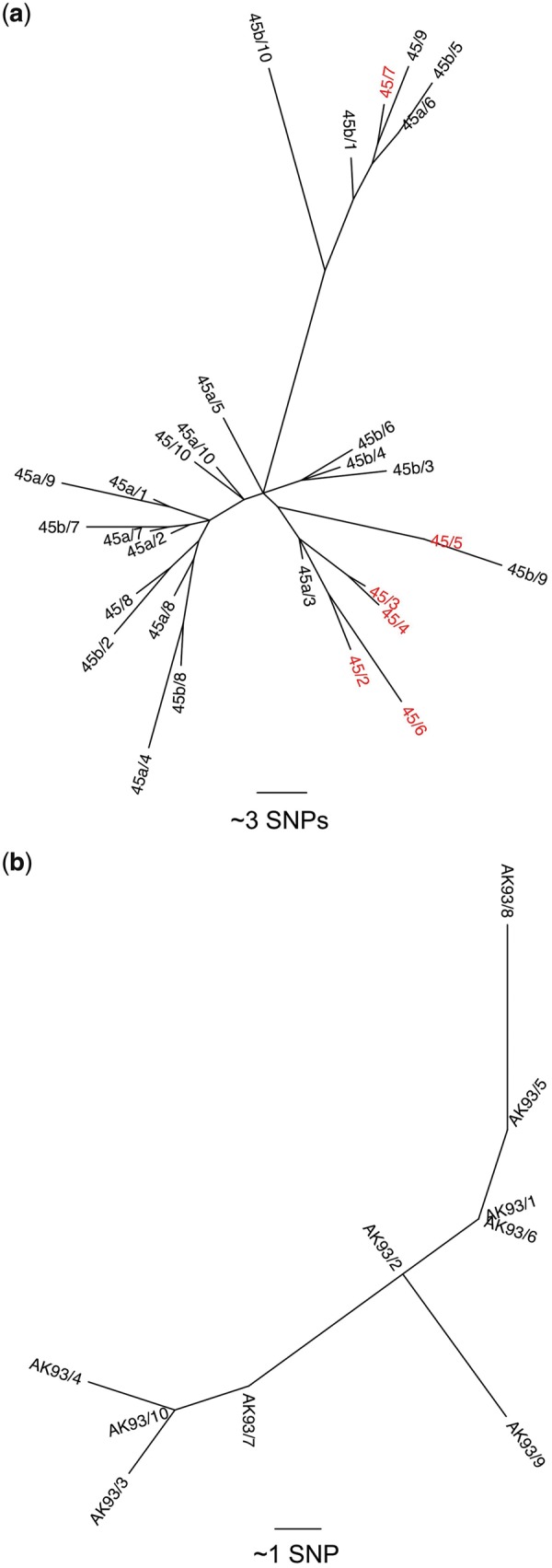
Multilocus sequence typing of both patients Patient 0045 carried the ST155 strain in all three samples (see Fig. 1a), and generation of a core genome phylogeny revealed that the 30 isolates differed by a total of 138 SNPs across the core genome. Patient 0093 carried a novel ST that was subsequently assigned as ST1025; the 10 isolates from this patient differed by a total of 13 SNPs (see Fig. 1b). ST: sequence type; SNP: single nucleotide polymorphisms.

*S. pseudintermedius* is a commensal and opportunistic pathogen of cats and dogs, in which it causes skin and soft tissue infections [4]. Increasingly, S*. pseudintermedius* is recognized as a zoonosis in humans [5, 6]. Re-analysis of isolates reported as *S. aureus* by clinical microbiology laboratories may identify *S. pseudintermedius* in a small proportion of patients [7]. A recent observational study at a large regional microbiology laboratory over a 2-year period reported the clinical characteristics of 24 patients who were culture-positive for *S. pseudintermedius* [5]. Most cases had severe co-morbidities and had contact with dogs at the time of infection (92.1%). Isolates were associated with skin and soft tissue infections in most cases (75%), although two patients had invasive disease [5]. This suggests that acquisition may occur from dogs, although a study that investigated the presence of *Staphylococcus* spp. in 119 dogs and their 107 owners found only one dog–owner pair that both carried *S. pseudintermedius* [8]. Neither patient in the present study had a history of contact with dogs, and thus it remains unclear which factors determine colonization with *S. pseudintermedius*.

To the best of our knowledge, this is the first evidence of persistent nasal carriage of *S. pseudintermedius* in humans. The detection of two distinct lineages demonstrates that colonization is not limited to a specific clone. Transmission and factors leading to persistent carriage are not known, but local damage relating to vasculitis and pharmacological immune suppression may make GPA patients more prone to colonization. It remains unclear whether *S. pseudintermedius* has any impact on relapse risk or is directly involved in the etiopathogenesis of GPA.

## Supplementary Material

Supplementary Data
