# Supplementary material for: Nasal carriage of Staphylococcus pseudintermedius in patients with granulomatosis with polyangiitis
Source: Rheumatology (Oxford). 2018 Nov 8;58(3):548–50. doi: 10.1093/rheumatology/key317 (PMC6381761; doi:10.1093/rheumatology/key317)
Supplement: Supplementary Data [file key317_supplementary_data.docx]

**SUPPLEMENTARY DATA**

**Supplementary Table 1. Demographics of both patients at the time of initial nasal swab.**

| Patient number | Age | Gender | Disease Duration, months | BVAS v3.0 | VDI | DEI | AB Treatment | Immunosuppression | ENT Assessment |
| --- | --- | --- | --- | --- | --- | --- | --- | --- | --- |
| 0045 | 68 | female | 168 | 0 | 1 | 2 | none | MMF | inactive |
| 0093 | 52 | male | 120 | 1 | 1 | 2 | none | rituximab, prednisolone (5 mg/day) | inactive |

Disease duration is given in months (between initial diagnosis and sampling). The respective study number of the patient is highlighted in the left column. We further highlighted whether our patients had concomitant antibiotic treatment, immunosuppression and performed a standardised investigation of disease scores (BVAS v3.0 and DEI), damage score (VDI) AND ENT assessment. AB: antibiotic; BVAS: Birmingham Vasculitis Activity Score; DEI: Disease Extent Index; VDI: Vasculitis Damage Index.

| **Supplementary table S2 Whole genome sequencing and sequence analysis Isolates** | | | | | | | | |  |  |
| --- | --- | --- | --- | --- | --- | --- | --- | --- | --- | --- |
| **Strain name** | **Sequence type (ST)** | **ack** | **cpn60** | **fdh** | **pta** | **purA** | **sar** | **tuf** | **Resistance genes** | **ENA accession** |
| 45/1 | 155 | 2 | 11 | 2 | 1 | 1 | 1 | 2 | tetM blaZ aacA-aphD | ERR1430755 |
| 45/2 | 155 | 2 | 11 | 2 | 1 | 1 | 1 | 2 | tetM blaZ aacA-aphD | ERR1430756 |
| 45/3 | 155 | 2 | 11 | 2 | 1 | 1 | 1 | 2 | tetM blaZ aacA-aphD | ERR1430757 |
| 45/4 | 155 | 2 | 11 | 2 | 1 | 1 | 1 | 2 | tetM blaZ aacA-aphD | ERR1430758 |
| 45/5 | 155 | 2 | 11 | 2 | 1 | 1 | 1 | 2 | tetM blaZ aacA-aphD | ERR1430759 |
| 45/6 | 155 | 2 | 11 | 2 | 1 | 1 | 1 | 2 | tetM dfrG blaZ aacA-aphD | ERR1430760 |
| 45/7 | 155 | 2 | 11 | 2 | 1 | 1 | 1 | 2 | tetM blaZ aacA-aphD | ERR1430761 |
| 45/8 | 155 | 2 | 11 | 2 | 1 | 1 | 1 | 2 | tetM dfrG blaZ aacA-aphD | ERR1430762 |
| 45/9 | 155 | 2 | 11 | 2 | 1 | 1 | 1 | 2 | tetM blaZ aacA-aphD | ERR1430763 |
| 45/10 | 155 | 2 | 11 | 2 | 1 | 1 | 1 | 2 | tetM blaZ aacA-aphD | ERR1430764 |
| 45a/1 | 155 | 2 | 11 | 2 | 1 | 1 | 1 | 2 | tetM blaZ aacA-aphD | ERR1430765 |
| 45a/2 | 155 | 2 | 11 | 2 | 1 | 1 | 1 | 2 | tetM blaZ aacA-aphD | ERR1430766 |
| 45a/3 | 155 | 2 | 11 | 2 | 1 | 1 | 1 | 2 | tetM blaZ aacA-aphD | ERR1430767 |
| 45a/4 | 155 | 2 | 11 | 2 | 1 | 1 | 1 | 2 | tetM blaZ aacA-aphD | ERR1430768 |
| 45a/5 | 155 | 2 | 11 | 2 | 1 | 1 | 1 | 2 | tetM blaZ aacA-aphD | ERR1430769 |
| 45a/6 | 155 | 2 | 11 | 2 | 1 | 1 | 1 | 2 | tetM dfrG blaZ aacA-aphD | ERR1430770 |
| 45a/7 | 155 | 2 | 11 | 2 | 1 | 1 | 1 | 2 | tetM blaZ aacA-aphD | ERR1430771 |
| 45a/8 | 155 | 2 | 11 | 2 | 1 | 1 | 1 | 2 | tetM blaZ aacA-aphD | ERR1430772 |
| 45a/9 | 155 | 2 | 11 | 2 | 1 | 1 | 1 | 2 | tetM blaZ aacA-aphD | ERR1430773 |
| 45a/10 | 155 | 2 | 11 | 2 | 1 | 1 | 1 | 2 | tetM blaZ aacA-aphD | ERR1430774 |
| 45b/1 | 155 | 2 | 11 | 2 | 1 | 1 | 1 | 2 | tetM dfrG blaZ aacA-aphD | ERR1430775 |
| 45b/2 | 155 | 2 | 11 | 2 | 1 | 1 | 1 | 2 | tetM blaZ aacA-aphD | ERR1430776 |
| 45b/3 | 155 | 2 | 11 | 2 | 1 | 1 | 1 | 2 | tetM blaZ aacA-aphD | ERR1430777 |
| 45b/4 | 155 | 2 | 11 | 2 | 1 | 1 | 1 | 2 | tetM blaZ aacA-aphD | ERR1430778 |
| 45b/5 | 155 | 2 | 11 | 2 | 1 | 1 | 1 | 2 | tetM dfrG blaZ aacA-aphD | ERR1430779 |
| 45b/6 | 155 | 2 | 11 | 2 | 1 | 1 | 1 | 2 | tetM blaZ aacA-aphD | ERR1430780 |
| 45b/7 | 155 | 2 | 11 | 2 | 1 | 1 | 1 | 2 | tetM blaZ aacA-aphD | ERR1430781 |
| 45b/8 | 155 | 2 | 11 | 2 | 1 | 1 | 1 | 2 | tetM blaZ aacA-aphD | ERR1430782 |
| 45b/9 | 155 | 2 | 11 | 2 | 1 | 1 | 1 | 2 | tetM blaZ aacA-aphD | ERR1430783 |
| 45b/10 | 155 | 2 | 11 | 2 | 1 | 1 | 1 | 2 | tetM dfrG blaZ aacA-aphD | ERR1430784 |
| AK93/1 | 1025 | 5 | 2 | 2 | 1 | 6 | 2 | 1 | blaZ | ERR1430785 |
| AK93/2 | 1025 | 5 | 2 | 2 | 1 | 6 | 2 | 1 | blaZ | ERR1430786 |
| AK93/3 | 1025 | 5 | 2 | 2 | 1 | 6 | 2 | 1 | blaZ | ERR1430787 |
| AK93/4 | 1025 | 5 | 2 | 2 | 1 | 6 | 2 | 1 | blaZ | ERR1430788 |
| AK93/5 | 1025 | 5 | 2 | 2 | 1 | 6 | 2 | 1 | blaZ | ERR1430789 |
| AK93/6 | 1025 | 5 | 2 | 2 | 1 | 6 | 2 | 1 | blaZ | ERR1430790 |
| AK93/7 | 1025 | 5 | 2 | 2 | 1 | 6 | 2 | 1 | blaZ | ERR1430791 |
| AK93/8 | 1025 | 5 | 2 | 2 | 1 | 6 | 2 | 1 | blaZ | ERR1430792 |
| AK93/9 | 1025 | 5 | 2 | 2 | 1 | 6 | 2 | 1 | blaZ | ERR1430793 |
| AK93/10 | 1025 | 5 | 2 | 2 | 1 | 6 | 2 | 1 | blaZ | ERR1430794 |

DNA was extracted and amplified from nasal swabs using the QIAamp DNA Microbiome Kit (QIAGEN, Hilden, Germany).

llumina library preparation was carried out as previously described [1] and Hi-seq sequencing was carried out following the manufacturer’s standard protocols (Illumina, Inc.). Nucleotide sequences been deposited in the European Nucleotide Archive (supplementary table S2, available at *Rheumatology* online). Fastq files were mapped against the *S. pseudintermedius* genome ED99 [2] using SMALT ([www.sanger.ac.uk/resources/software/smalt/](http://www.sanger.ac.uk/resources/software/smalt/)) in order to identify SNPs. SNPs located in MGEs were removed from the alignment.  Maximum likelihood trees were generated from these core genome SNPs using RAxML [3]. Trees were visualized and annotated using FigTree. Sequence data were assembled using a previously described pipeline [4]. For each isolate the sequence reads were used to create multiple assemblies using VelvetOptimiser v2.2.5 [5] and Velvet v1.2 [6]. The assemblies were improved by scaffolding the best N50 and contigs using SSPACE [7] and sequence gaps filled using GapFiller [8]. Multilocus sequence types (MLST) were determined from the assemblies using MLST check (https://github.com/sanger-pathogens/mlst_check), which was used to compare the assembled genomes against the MLST database for *S. pseudintermedius* (<https://pubmlst.org/spseudintermedius/>) [9]. The presence antibiotic resistance genes were identified using BLAST against the assemblies.

**Supplementary Figure S1. Colonies obtained from patients with granulomatosis with polyangiitis undergoing nasal swab examination**


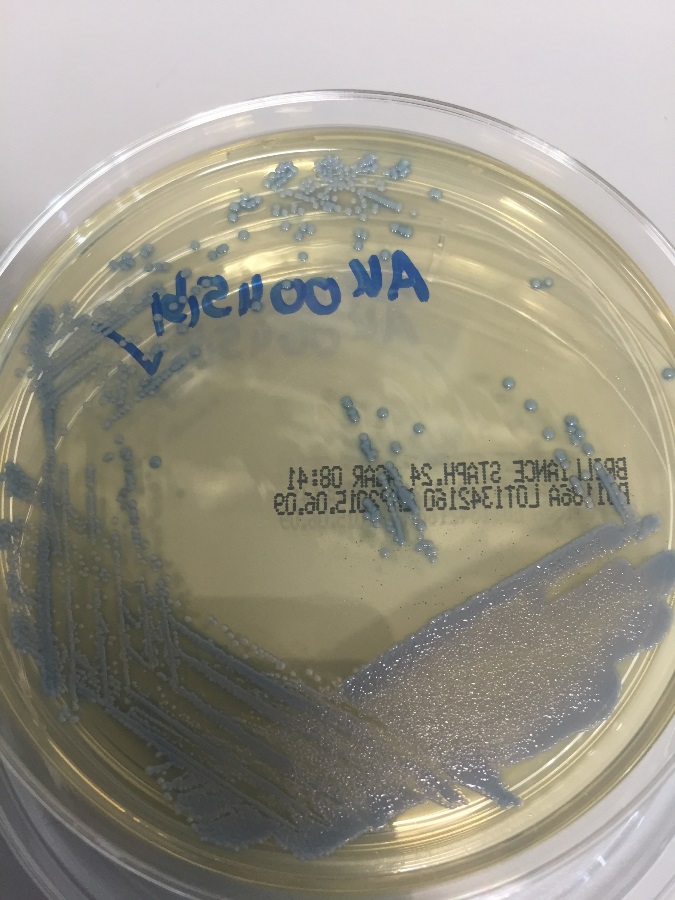

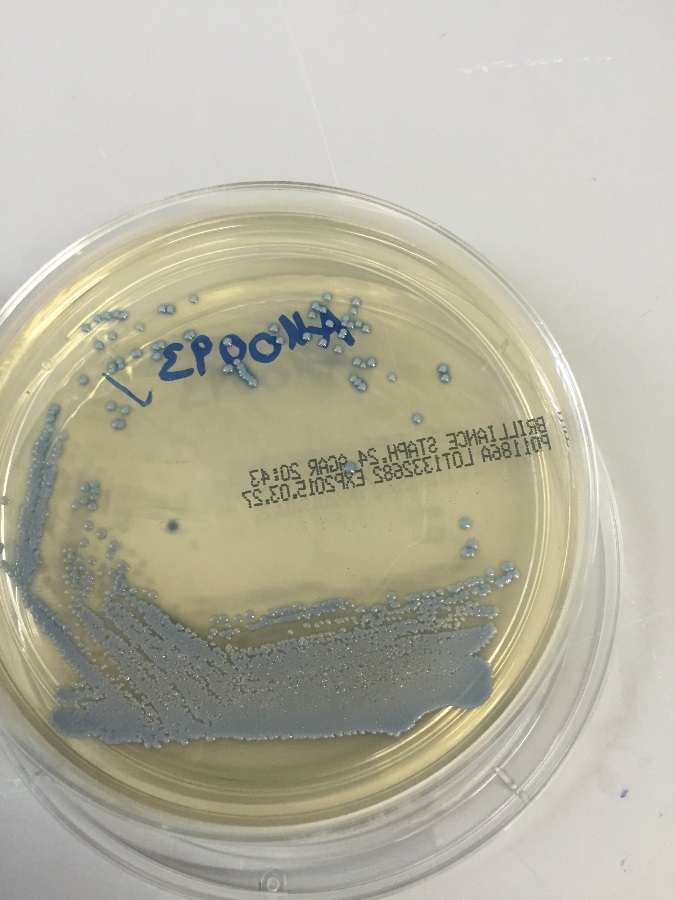


**B**

**A**


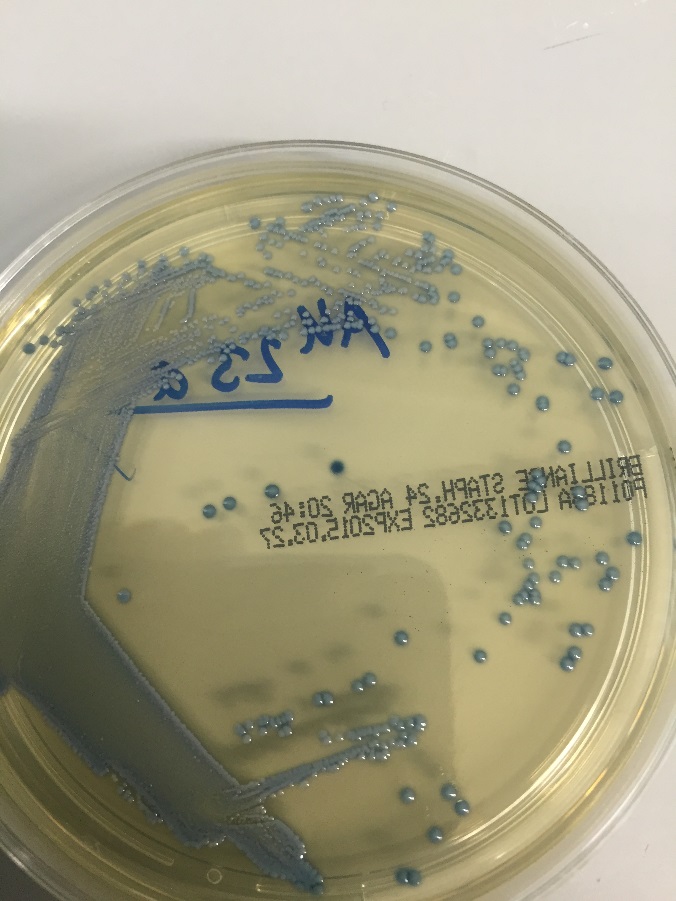

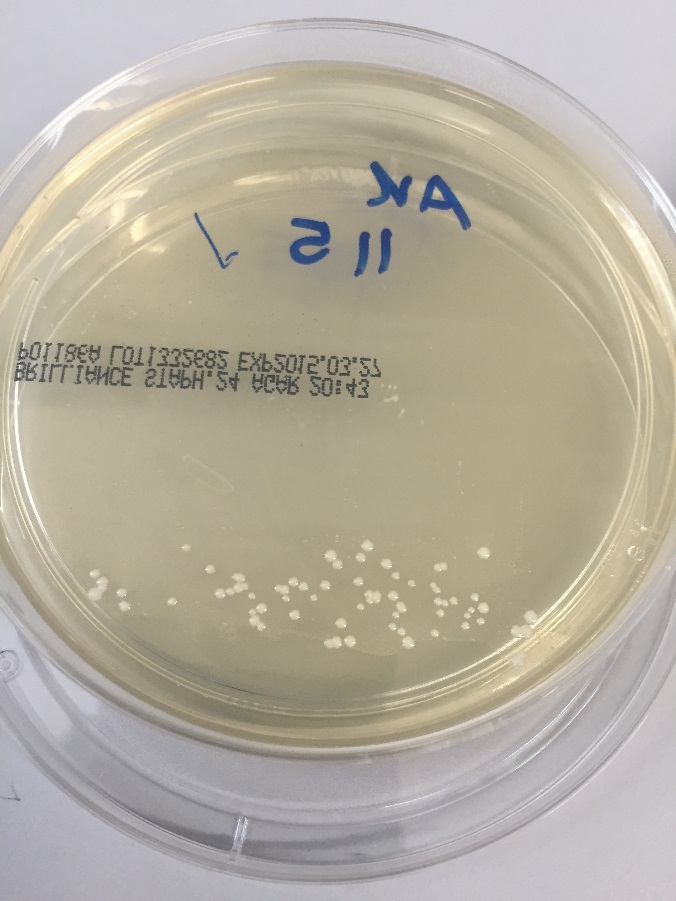


**D**

**C**

Respective colonies obtained from Brilliance Staph 24 Agar plates (Oxoid, United Kingdom) were identified by matrix assisted laser desorption ionization - time of flight mass spectrometry (MALDI-TOF MS). A) and B) revealed presence of Staphylococcus pseudintermedius, while C) and D) identified Staphylococcus aureus and Staphylococcus epidermidis, respectively.

**References**

1 Quail MA, Kozarewa I, Smith F, et al. A large genome center's improvements to the Illumina sequencing system. Nature methods 2008;5(12):1005-10.

2 Zakour NLB, Bannoehr J, van den Broek AHM, Thoday KL, Fitzgerald JR. Complete Genome Sequence of the Canine Pathogen Staphylococcus pseudintermedius. Journal of bacteriology 2011;193(9):2363-4.

3 Stamatakis A, Ludwig T, Meier H. RAxML-III: a fast program for maximum likelihood-based inference of large phylogenetic trees. Bioinformatics 2005;21(4):456-63.

4 Page AJ, De Silva N, Hunt M, et al. Robust high throughput prokaryote de novo assembly and improvement pipeline for Illumina data. bioRxiv 2016.

5 Gladman S, Seemann T, Consortium VB. Velvet Optimiser: For automatically optimising the primary parameter options for the Velvet de novo sequence assembler. . 2008.

6 Zerbino DR, Birney E. Velvet: algorithms for de novo short read assembly using de Bruijn graphs. Genome Res 2008;18(5):821-9.

7 Boetzer M, Henkel CV, Jansen HJ, Butler D, Pirovano W. Scaffolding pre-assembled contigs using SSPACE. Bioinformatics 2011;27(4):578-9.

8 Boetzer M, Pirovano W. Toward almost closed genomes with GapFiller. Genome Biol 2012;13(6):R56.

9 Solyman SM, Black CC, Duim B, et al. Multilocus sequence typing for characterization of *Staphylococcus pseudintermedius*. Journal of clinical microbiology 2013;51(1):306-10.
